# Dose- and Ion-Dependent Effects in the Oxidative Stress Response to Space-Like Radiation Exposure in the Skeletal System

**DOI:** 10.3390/ijms18102117

**Published:** 2017-10-10

**Authors:** Joshua S. Alwood, Luan H. Tran, Ann-Sofie Schreurs, Yasaman Shirazi-Fard, Akhilesh Kumar, Diane Hilton, Candice G. T. Tahimic, Ruth K. Globus

**Affiliations:** 1 Bone and Signaling Laboratory, Space BioSciences Division, NASA Ames Research Center, Mail-Stop 236-7, Moffett Field, CA 94035, USA; joshua.s.alwood@nasa.gov (J.S.A.); luan.h.tran@nasa.gov (L.H.T.); ann-sofie.schreurs@nasa.gov (A.-S.S.); yasaman.shirazi-fard@nasa.gov (Y.S.-F.); akhilesh482@gmail.com (A.K.); Hilton.Diane@att.net (D.H.); candiceginn.t.tahimic@nasa.gov (C.G.T.T.); 2 Wyle Laboratories, Mail-Stop 236-7, Moffett Field, CA 94035, USA

**Keywords:** cancellous bone, osteoblast, ionizing radiation, spaceflight, oxidative stress

## Abstract

Space radiation may pose a risk to skeletal health during subsequent aging. Irradiation acutely stimulates bone remodeling in mice, although the long-term influence of space radiation on bone-forming potential (osteoblastogenesis) and possible adaptive mechanisms are not well understood. We hypothesized that ionizing radiation impairs osteoblastogenesis in an ion-type specific manner, with low doses capable of modulating expression of redox-related genes. 16-weeks old, male, C57BL6/J mice were exposed to low linear-energy-transfer (LET) protons (150 MeV/n) or high-LET ^56^Fe ions (600 MeV/n) using either low (5 or 10 cGy) or high (50 or 200 cGy) doses at NASA’s Space Radiation Lab. Five weeks or one year after irradiation, tissues were harvested and analyzed by microcomputed tomography for cancellous microarchitecture and cortical geometry. Marrow-derived, adherent cells were grown under osteoblastogenic culture conditions. Cell lysates were analyzed by RT-PCR during the proliferative or mineralizing phase of growth, and differentiation was analyzed by imaging mineralized nodules. As expected, a high dose (200 cGy), but not lower doses, of either ^56^Fe or protons caused a loss of cancellous bone volume/total volume. Marrow cells produced mineralized nodules ex vivo regardless of radiation type or dose; ^56^Fe (200 cGy) inhibited osteoblastogenesis by more than 90% (5 weeks and 1 year post-IR). After 5 weeks, irradiation (protons or ^56^Fe) caused few changes in gene expression levels during osteoblastogenesis, although a high dose ^56^Fe (200 cGy) increased *Catalase* and *Gadd45*. The addition of exogenous superoxide dismutase (SOD) protected marrow-derived osteoprogenitors from the damaging effects of exposure to low-LET (^137^Cs γ) when irradiated in vitro, but had limited protective effects on high-LET ^56^Fe-exposed cells. In sum, either protons or ^56^Fe at a relatively high dose (200 cGy) caused persistent bone loss, whereas only high-LET ^56^Fe increased redox-related gene expression, albeit to a limited extent, and inhibited osteoblastogenesis. Doses below 50 cGy did not elicit widespread responses in any parameter measured. We conclude that high-LET irradiation at 200 cGy impaired osteoblastogenesis and regulated steady-state gene expression of select redox-related genes during osteoblastogenesis, which may contribute to persistent bone loss.

## 1. Introduction

Structural degradation and oxidative stress following exposure to space radiation potentially endangers skeletal health of astronauts, both during the mission and later in life. Weightlessness-induced musculoskeletal atrophy, psychological stress, and confinement pose additional risks to astronaut health [1]. For exploration missions into deep space, galactic cosmic-rays (GCR) are of particular concern because of the high-energy and densely ionizing pattern of energy deposition from heavy ions [2,3,4], which can induce oxidative stress [5,6] in various tissues. Both solar particle-emissions (SPE) and GCR contribute to the mixed composition of low-energy radiation (such as protons and γ rays), high linear-energy-transfer (LET) particles (such as ionized ^56^Fe- and ^12^C-nuclei), and secondary constituents, such as Bremmstrahlung, fragmentation products, and neutrons [4,7]. Estimates of the total absorbed dose during a Mars mission (180-day transits with 500-day surface operations) to be between 28–47 cGy, with GCR contributing 27 cGy [8,9]. Currently, risk of exposure-induced death from a fatal cancer drives the NASA astronaut career radiation-exposure limit [10], though NASA is investigating additional risks associated with acute and late tissue degeneration following radiation exposure [11].

Irradiation perturbs the redox balance within the bone and marrow compartment in a complex and transient manner. Total-body irradiation (^137^Cs, 200 cGy) induces rapid reactive oxidative species formation in the marrow [12] and increases expression of the master transcription factor that binds to the antioxidant response element, nuclear factor (erythroid-derived 2)-like 2 (*Nrf2)* [13]. Excess reactive oxygen species (ROS) can interfere with osteogenesis of bone marrow derived osteoprogenitors [14,15] as well as contribute to bone resorption by osteoclasts [16]. High-LET ^56^Fe (≥200 cGy) impairs cell proliferation during osteoblastogenesis from marrow progenitors in a dose-dependent manner [17]. Further, irradiation in vitro with ^56^Fe (100 cGy) arrests the cell cycle and inhibits proliferation of mesenchymal stem cells [18]. At higher doses (400–800 cGy), irradiation with X-rays increases ROS, depletes antioxidant stores, increases Nrf2 protein levels, and reduces differentiation in osteoblast-like cells [19]. However, the role of oxidative stress on the potential for total-body irradiation (TBI) to impair osteoblastogenesis over the long term, in particular low-dose, high-LET species, has not been fully characterized.

We posed three principal questions to address in this study. First, are the severity of acute bone loss [12,13,20,21] and impairment of osteoblastogenesis [17] following TBI dependent on radiation type (LET) or dose? Secondly, which pathways are activated? Finally, do irradiation-induced insults to skeletal structure and osteoblastogenesis persist long after exposure? We hypothesized that: (1) irradiation induces bone loss, shown previously to be dominated by total dose of exposure [22], and leads to temporal perturbations in antioxidant defenses; (2) TBI induces both persistent changes in redox-related genes and cancellous microarchitecture in an ion-dependent manner; and, (3) addition of an antioxidant, here the enzyme superoxide dismutase (SOD), mitigates irradiation-induced damage to osteoprogenitors and stem cells when exposed in vitro, implicating an oxidative-stress dependent mechanism.

To address these questions, we conducted in vivo and in vitro experiments. For in vivo work, we selected protons and ^56^Fe ions as representative radiation for the abundant low-LET particles in space radiation and the heavy-ion component of galactic cosmic radiation, respectively. For in vitro work, we selected ^137^Cs γ rays as a low-LET reference radiation to enable high-throughput countermeasure testing. In vivo, there were persistent and profound decrements in mineralized nodules after TBI (at 200 cGy) with ^56^Fe, while protons had a milder effect. In contrast to our hypothesis, few changes were observed in the steady-state expression of redox-related genes during ex vivo osteoblastogenesis following TBI, although there was some indication of altered expression of select genes during the proliferative phase (at 200 cGy ^56^Fe, but not lower doses). Furthermore, scavenging ROS with an antioxidant (i.e., SOD) mitigated adverse effects of in vitro irradiation with γ rays, but failed to protect fully from irradiation with an equivalent dose of heavy ions.

## 2. Results

### 2.1. TBI Experiment Design

**Figure 1:**
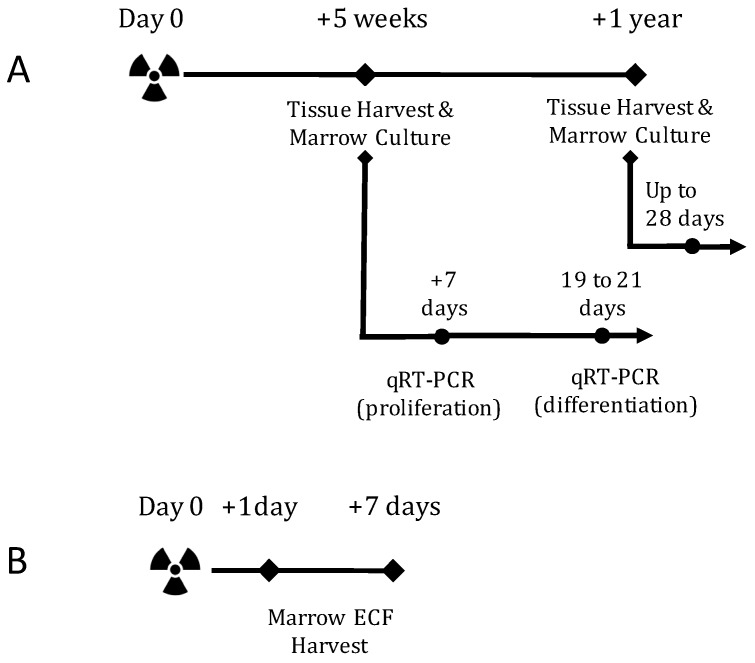
Total Body Irradiation (TBI) experiment designs. (**A**) Experiments to determine whether TBI alters antioxidant gene expression in osteoblasts, osteoblastogenesis, and the structure of cancellous bone in an ion-dependent fashion at five weeks after proton or ^56^Fe exposure and to determine the osteoblastogenic and structural sequelae of ^56^Fe irradiation one year after exposure. At each endpoint, marrow cells were cultured for osteoblastogenesis. At 5 weeks post-irradiation, osteoblastogenic cultures were halted during the proliferative phase and differentiation phase for gene expression assessment; (**B**) Experiment to determine the acute effect of proton or ^56^Fe irradiation on the antioxidant status of the bone marrow extracellular fluid (ECF). Mice were irradiated and euthanized either one or seven days after exposure. Marrow was collected and cells removed through centrifugation. The supernatant, defined as the extracellular fluid (ECF), was assessed for total antioxidant capacity.

To evaluate the individual effects of radiation type, our study queried a dose response to high-LET ^56^Fe or low-LET protons (see Methods below for details) at one day, seven days, or five weeks post-exposure. Additionally, we assessed the sequelae of ^56^Fe irradiation at a late, 1-year post exposure endpoint (Figure 1A,B). Tibiae and femora were collected for assessment of the cancellous microarchitecture. Bone-marrow cells were cultured ex vivo in osteoblastogenic media for colony counts and mineralization assays. Additionally, the effects of total body irradiation on gene expression were assayed at two points, during the proliferation and mineralization phase, of osteoblastogenesis.

### 2.2. Body Mass Temporal Responses and Coat Color

**Figure 2:**
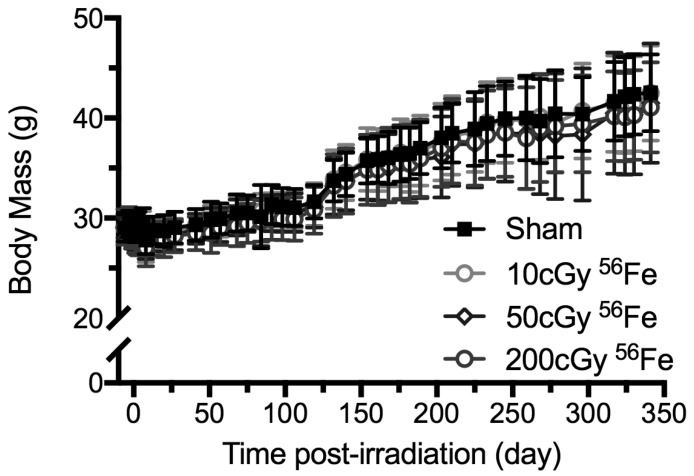
Longitudinal body mass measurements of ^56^Fe-irradiated mice up to 1 year post-exposure (mice from Figure 1A). Mice were irradiated on day 0 and shipping took place between days 6 and 8. Data are mean ± standard deviation with *n* = 13 per group.

Body mass was measured (±0.1 g), at least weekly, throughout the study as an index of general health (Figure 2, showing the 1-year post irradiation cohorts). As determined by one-way analysis of variance (ANOVA) and Dunnett’s posthoc, within six days of exposure, ^56^Fe (200 cGy) reduced the cohort’s mean body mass by 4% relative to the starting weights. Other doses and ions had no significant effect relative to starting weights [23]. Shipment of the mice from Brookhaven National Lab (BNL) to NASA Ames between days 6 and 8, caused a 5% decline in body weights of all of the groups.

Following shipment, the body mass of all groups rose over time (+7% for the 5-weeks endpoint and +46% for the 1-year endpoint), with no treatment effect, nor interaction of time × treatment (Figure 2). By the time of euthanasia at 5-weeks or 1-year post-irradiation, body mass did not significantly differ between irradiated and age-matched sham-controls.

Additionally, as an index of general health, the coat color was assessed as grey or black at the end of the experiment (1-year post-irradiation). In sham controls, 1 out of 13 mice had a grey coat (Table 1). In contrast, ^56^Fe (200 cGy) caused all of the 11 to have grey coats. The lower doses of ^56^Fe irradiation, 10 or 50 cGy, did not modulate coat color compared to sham-controls, respectively.

### 2.3. Bone Structure

**Figure 3:**
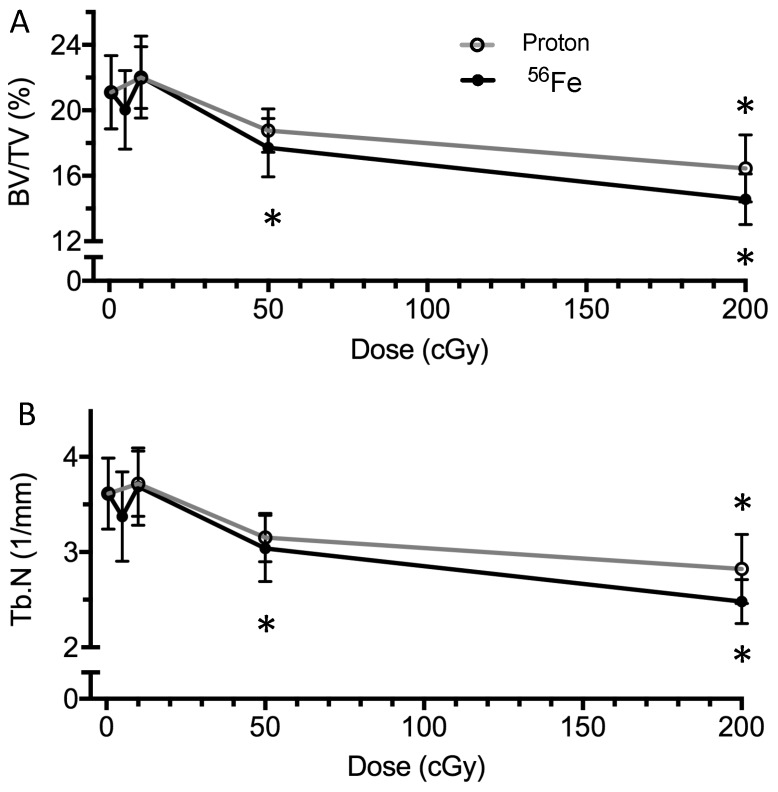
Dose response of cancellous bone microarchitecture following proton or ^56^Fe irradiation at 5 weeks after exposure (mice from Figure 1A). Microarchitecture was assessed using microcomputed tomography and the parameters of (**A**) bone volume / total volume (BV/TV, %) and (**B**) trabecular number (Tb.N, 1/mm). Data are mean ± standard deviation, with *n* = 8/group. * denotes *p* ≤ 0.05 vs. age-matched sham control.

Microcomputed tomography was used to assess the effects of aging and irradiation on the cancellous tissue in the proximal tibial metaphysis at the 5 week and 1 year endpoints (Figure 1A) and cortical geometry of the femoral midshaft at the 1-year endpoint. In sham-treated animal cohorts, aging caused a 54% reduction of cancellous bone volume/total volume (BV/TV) between the 5-week (Figure 3A, 21.1 ± 2.2%) and 1-year (Table 1, 9.7 ± 3.1%) post-treatment endpoints.

At 5 weeks post-irradiation, 50 and 200 cGy ^56^Fe caused a decrement in BV/TV (Figure 3A, −16% and −31%, respectively) and strut number (Tb.N, Figure 3B, −16% and −31%, respectively) in the cancellous region of the proximal tibia, compared to shams. For protons, 200 cGy caused a −22% decrement in BV/TV (Figure 3A) and Tb.N (Figure 3B), while 50 cGy showed a trend towards decreased BV/TV (−11%, *p* = 0.12) and Tb.N (−13%, *p* = 0.06). Additionally, irradiation at 200 cGy increased trabecular separation, but did not affect trabecular thickness [23]. At 5 or 10 cGy, neither ^56^Fe, nor protons, showed any detectable bone loss, by any structural measure.

At 1 year post-irradiation, ^56^Fe (200 cGy) showed a trend of decreased BV/TV and Tb.N (−25%, *p* = 0.12, Table 1) in the cancellous region of the proximal tibia, as compared to age-matched controls. No changes were observed at 10 or 50 cGy doses when compared to controls. Additionally, no significant changes in cortical geometry of the femur at midshaft (bone volume and cortical thickness, [23]) were detected, as compared to age-matched controls.

### 2.4. Ex Vivo Osteoblastogenesis

To assess the cellular responses to irradiation during ex vivo osteoblast growth and maturation, we cultured marrow progenitor cells at the 5 week and 1 year post-irradiation endpoints. For cultures plated 5 weeks post-irradiation, gene expression, alkaline phosphatase activity, colony counts, and DNA content analyses were performed during the proliferative phase (seven days after plating) and mineralization phase (19 to 21 days after plating). At the end of the latter phase, the mineralized area was quantified to assess the functional outcome of differentiation.

**Figure 4:**
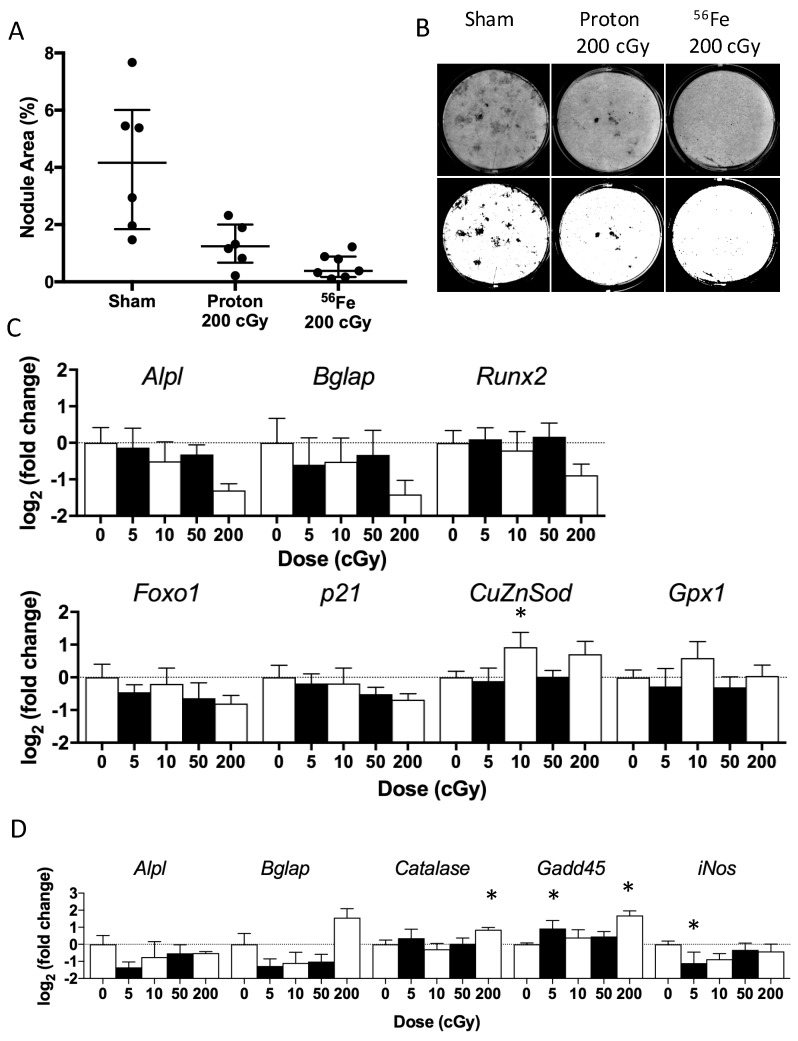
Differentiation and proliferation responses of osteoprogenitors cultured from bone marrow of mice that received proton or ^56^Fe irradiation (5 weeks post-irradiation from mice in Figure 1A). (**A**) Nodule area of cultures from animals irradiated with 200 cGy proton or ^56^Fe compared to sham control. Data are summarized by median and interquartile range with *n* = 6–7/group. Each dot represents an individual mouse. (**B**) Grayscale (top row) and binarized (bottom row) images of representative wells (i.e., near the group median), showing nodules during the mineralization phase for 200 cGy proton or iron or sham. Gene expression assessed during (**C**) terminal differentiation or (**D**) proliferation for markers of differentiation, damage response, cell cycle, and redox response in cells cultured from the marrow of previously irradiated mice. Data are mean ± standard deviation of Log_2_ (Fold Change) transformed values relative to the endogenous gene *HPRT* and normalized to the sham control (i.e., ΔΔ*C*_t_). The dashed line at 1.0 indicates the sham control level. *n* = 4/group, * denotes *p* ≤ 0.05 vs. sham-control.

During the mineralization phase, we observed well-formed nodules in the sham and proton-irradiated (200 cGy) groups (Figure 4A,B), with sham values displaying wide variance. In contrast, we observed very few, yet fully mineralized, nodules in the ^56^Fe-irradiated (200 cGy) group (Figure 4A,B). After quantifying the nodule area, irradiation with ^56^Fe (200 cGy) tended to cause a −91% decrement (*p* = 0.06, Figure 4A,B) in the mineralization area (median) as compared to sham-irradiated controls. This inhibitory effect is consistent with findings from our other experiments with this dose of ^56^Fe at various times after exposure [23]. Although, mineralization area of cultures from animals exposed to protons (200 cGy) tended to be lower than sham-controls (−70%, *p* = 0.10, Figure 4A,B), nodules from proton-irradiated mice appeared larger and more widespread than in cultures from ^56^Fe-irradiated mice (Figure 4B). Lower doses of either species did not affect mineralized area or any other measured parameters [23].

Radiation exposure did not modify expression levels for most of the genes analyzed during the mineralization phase (Table 2), although a high dose of ^56^Fe (200 cGy) tended to decrease expression of the osteoblast differentiation gene *Alpl* by −60% (*p* = 0.12), Figure 4C. Additionally, a low dose of ^56^Fe (10 cGy) increased the expression of the antioxidant gene *CuZnSOD* (+90%) as compared to sham-controls, whereas proton exposure did not modify expression levels for any of the genes assayed [23]. Taken together, these data suggest mild effects late in culture, but only for ^56^Fe, not protons.

During the proliferative phase, most doses and types of radiation exposure did not modify the expression levels for the genes analyzed when compared to sham-controls (Table 2). A high-dose of ^56^Fe (200 cGy) did however increase the expression levels of genes for the cell cycle arrest marker *Gadd45* (+223%) and the antioxidant *Catalase* (+81%), and also tended to increase the late-osteoblastic marker *Bglap* (+196%, *p* = 0.08) (Figure 4D). A lower dose of ^56^Fe (5 cGy) decreased the gene expression of the nitric oxide generator *iNOS* by −53% and increased Gadd45 by +91%, respectively, when compared to sham controls.

**Figure 5:**
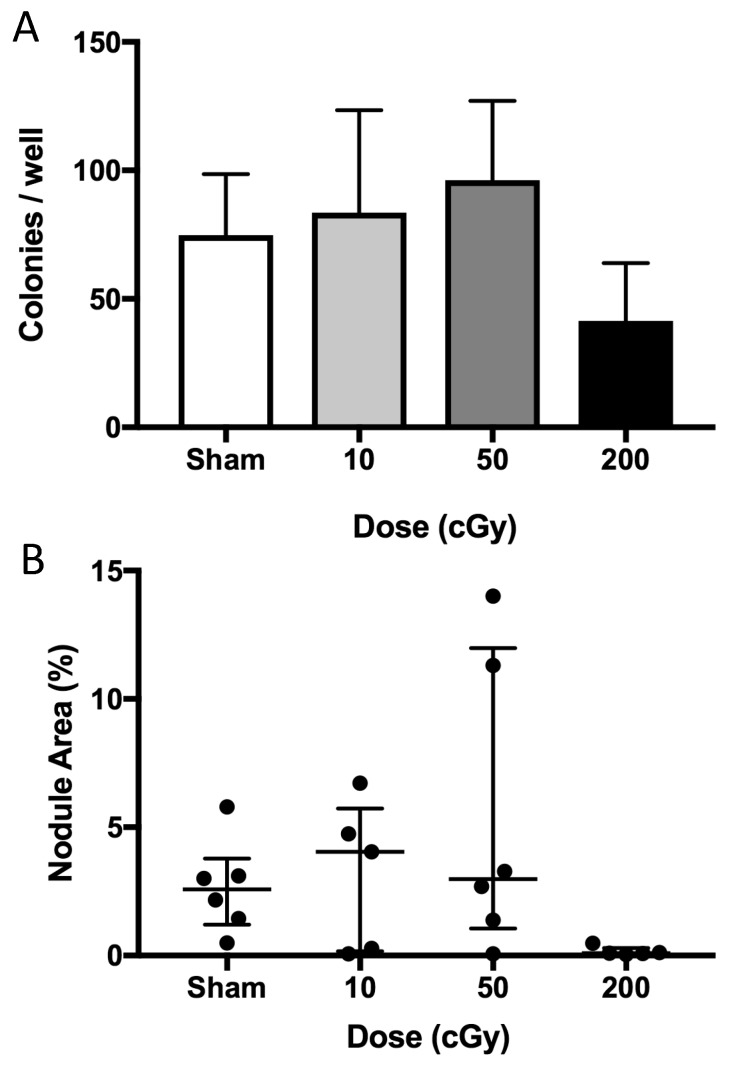
Persistent effects of ^56^Fe (200 cGy) TBI on osteoblastogenesis at 1 year post-exposure (mice from Figure 1A). Marrow was cultured in osteoblastogenic conditions. We quantified (**A**) colony number on day 8 in vitro and (**B**) nodule area on day 28 in vitro (with dots representing individual mice). Data are summarized by mean and standard deviation (**A**) and median and interquartile range (**B**), with *n* = 5–6/group.

For the ex vivo marrow culture performed 1-year post irradiation, the numbers of colonies were counted eight days after plating and nodule areas were assessed 28 days after plating. ^56^Fe irradiation (200 cGy) tended to reduce colony counts at day eight (−45%, Figure 5A) when compared to controls, whereas lower doses of ^56^Fe (10 and 50 cGy) had no effect. At 28 days after plating, cells from ^56^Fe (200 cGy)-irradiated mice tended to show reduced mineralized area (−96% reduction of median, *p* < 0.06, Figure 5B), whereas lower doses had no effect as compared to controls. Taken together, these data suggest that only the highest dose of ^56^Fe had a persistent effect to inhibit osteoblastogenesis, as assessed by colony formation.

### 2.5. Total Antioxidant Capacity of the Marrow Extracellular Fluid

**Figure 6:**
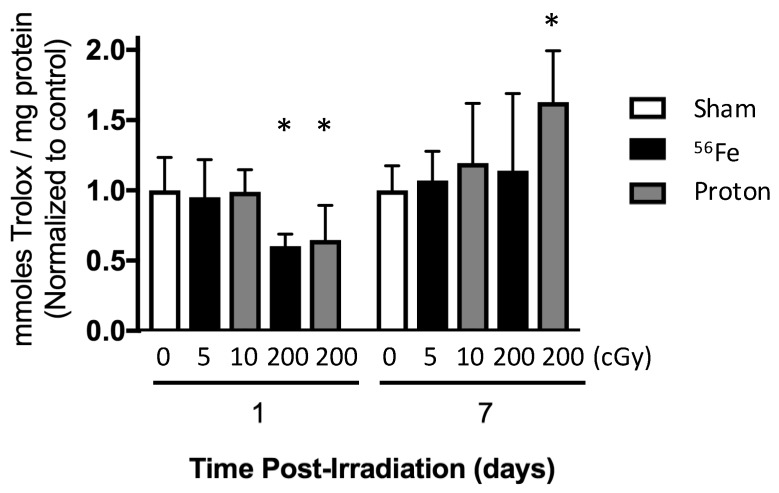
Irradiation perturbation of the marrow oxidative milieu, assessed by the antioxidant capacity of the extracellular fluid (ECF), in mice at one and seven days post exposure (following timeline in Figure 1B). Mice were irradiated with ^56^Fe at 5 or 200 cGy or protons at 10 or 200 cGy. Data are mean ± standard deviation. * *p* ≤ 0.05 versus sham control.

To assess the changes in the redox microenvironment in the marrow cavity following irradiation, we measured the antioxidant capacity of the extracellular fluid from the marrow, (normalized by protein concentration) at one and seven days after exposure to ^56^Fe or protons (Figure 1B). Protons (200 cGy) or ^56^Fe (200 cGy) decreased the total antioxidant capacity of the marrow extracellular fluid by over 35% within one day of exposure (Figure 6). In contrast, seven days after exposure, protons caused a 62% elevation of antioxidant capacity (Figure 6), whereas ^56^Fe did not differ from sham controls. Lower doses of radiation (5 cGy ^56^Fe or 10 cGy proton), did not modify antioxidant capacity per proton concentration with sham-controls at either timepoint.

### 2.6. In Vitro Radiation Effects on Osteoblastogenesis

**Figure 7:**
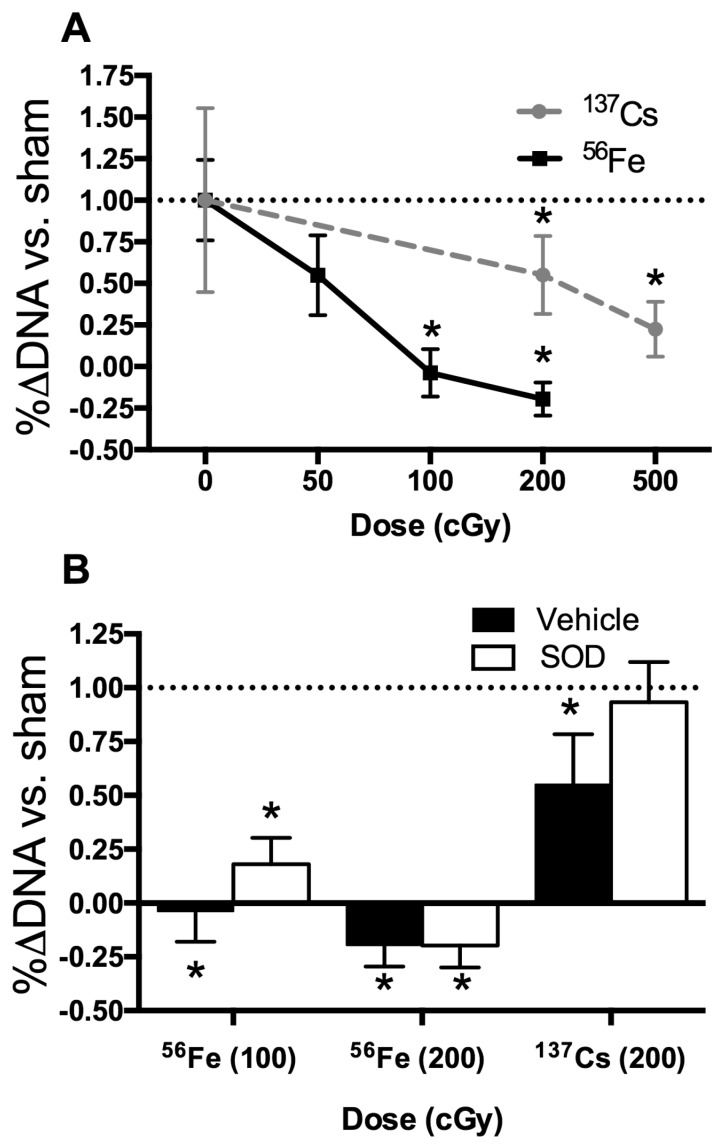
In vitro irradiation effects on osteoblastogenesis. (**A**) Dose response curve of osteoprogenitor growth after exposure to radiation using low-LET ^137^Cs or high-LET ^56^Fe; (**B**) Addition of the antioxidant superoxide dismutase (SOD) and its prevention of the irradiation-induced decrement in cell growth. Data are shown normalized to the sham control (which is depicted by the dashed line at 1.0), expressed as mean ± standard deviation. * denotes *p* ≤ 0.05 versus the sham-irradiated control.

We hypothesized that a radiation-induced rise in ROS damages osteoprogenitors, leading to a decrease in number and activity of differentiated progeny and therefore exogenous antioxidant is expected to mitigate adverse effects of radiation on the proliferation and subsequent differentiation of osteoprogenitors derived from the marrow. The influence of a low-LET species (^137^Cs) was compared to that of high-LET ^56^Fe. Adherent marrow cells grown under osteoblastogenic culture conditions were irradiated at day 3 in culture and the percentage change in DNA content between day 3 (day of irradiation) and day 10 calculated as a surrogate of culture growth (proliferation). Analysis of the dose-response to ^137^Cs and ^56^Fe revealed significant decrements in growth after exposure to ^137^Cs (200 cGy and 500 cGy) or ^56^Fe (100 cGy, 200 cGy), but not after exposure to lower doses (Figure 7A). Colony counts also were affected at the higher doses of radiation [23]. In summary, as shown in Figure 7A, osteoprogenitors appeared more sensitive to high-LET ^56^Fe than low-LET ^137^Cs γ.

To begin to assess the contribution of oxidative stress after in vitro radiation exposure of osteoprogenitors, several antioxidants (including SOD with and without polyethylene glycol (PEG); SOD with nanoparticles or liposomes; Catalase with and without PEG) were screened by addition to the culture media, with SOD (without carrier) showing the most promising results [23]. Addition of exogenous SOD (200 U/mL) provided twice a day (on day 3 and day 4) effectively protected cell growth from irradiation with ^137^Cs, whereas higher doses of SOD or longer periods of treatment were less effective [23]. As shown in Figure 7B, addition of SOD prevented the inhibition of growth by ^137^Cs (200 cGy). In contrast, addition of SOD was not as sufficient to rescue the cell death incurred after exposure to an equivalent dose of ^56^Fe (200 cGy).

## 3. Discussion

Heavy-ion irradiation during space missions is a risk to the skeletal health of astronauts, causing skeletal degeneration and cellular damage in simulations with rodents [12,20,24]. In this study, we sought to elucidate the role of radiation type and changes in the oxidative milieu of bone marrow related to marrow-derived osteoprogenitor differentiation. We found that low doses (below 50 cGy) of either low-LET protons or high-LET ^56^Fe did not cause observable adverse effects on structure and osteoblastogenesis at any endpoint, and, further, did not strongly perturb antioxidant capacity at seven days of exposure (Figure 6) nor expression levels of redox-related genes (Figure 4, Table 2) at 35 days of exposure. In contrast, only a high dose of ^56^Fe radiation (200 cGy) was sufficient to induce detrimental effects on total antioxidant capacity, bone structure, and osteoprogenitor populations of the marrow (Figure 3, Figure 4, Figure 5 and Figure 6). These changes were associated with transient elevations in mRNA levels (Figure 4C,D) of an antioxidant gene (*Catalase*) and a DNA-damage/cell cycle arrest marker (*Gadd45*) during ex vivo osteoblastogenesis, which we speculate indicates persistent oxidative stress or enhanced antioxidant defenses during the proliferative phase. Interestingly, cultures showed a trend towards elevation of osteocalcin (*Bglap*, Figure 4D), a late marker of differentiation, during the proliferative phase, which may indicate that ^56^Fe exposure accelerated differentiation.

Body mass and coat color were assessed as general indices of animal health. Body mass indicated that animals showed a modest and transient decline in response to ^56^Fe irradiation with 200 cGy (Figure 2), consistent with our previous studies [17]. Additionally, ^56^Fe irradiation at 200 cGy uniquely caused a shift in coat color from black to grey (Table 1). We interpret this as a hallmark of oxidative stress in the hair follicle [25], and, when coupled with the osteoprogenitor effects, this may signal premature aging in a second tissue, in line with the free-radical theory of cellular aging [26,27].

In rodents, radiation injury to bone between with ^56^Fe (10–200 cGy, 1 GeV/n) [17] manifests as acute cell death of marrow cells and concomitant cancellous bone loss driven by osteoclasts [12,22,28]. In this work, we showed that high-dose ^56^Fe or proton (200 cGy) caused a transient reduction in antioxidant capacity in the marrow microenvironment (Figure 6), potentially indicative of a depletion of extracellular antioxidant stores with subsequent recovery of the extracellular antioxidant defense system [29]. These findings are consistent with our data showing the marrow cell response to increase mRNA expression levels of the master antioxidant transcription factor, *Nrf2*, shown previously [13], and with effects also evident in a cell line at even higher doses [19]. Our group showed that a dietary supplement rich in antioxidants and polyphenols (dried plum) prevents bone loss and *Nrf2* induction from γ irradiation in mice [30], supportive of the role of oxidative stress in driving acute bone loss. Additionally, in vitro, 200 cGy ^56^Fe impairs osteoblastogenesis [17]. Our work extends this high-dose impairment in vivo and to five weeks and one year post-exposure, suggesting an irreversible insult to osteoblastogenesis.

Gene expression analyses during the proliferative phase (Figure 4D) revealed that a high dose of ^56^Fe (200 cGy) increased the expression of genes for the cell cycle arrest marker *Gadd45* and antioxidant *Catalase* relative to sham. These data are consistent with a G2/M arrest from high-LET irradiation of mesenchymal stem cells [18], a pre-osteoblast cell line [31], and many other cell types [32]. Interestingly, these two genes are targets of *Foxo* transcription factors [33,34,35]. Nrf2, Foxos, and p21 are critical to the antioxidant response element [36]. Oxidative stress activates Foxo transcription factors [37] and interferes with Wnt signaling [14,38,39,40]. Hence, our data suggest Foxo activation may play a role in ^56^Fe irradiation, decreasing osteoprogenitor cell proliferation, and, ultimately, mineralization levels.

During the mineralization phase (Figure 4A,B), ^56^Fe irradiation (200 cGy) tended to reduce nodule formation and alkaline phosphatase gene expression, suggesting a reduction in early markers of early-stage osteoblast differentiation and fewer cells contributing to nodule formation, consistent with in vitro evidence for high-dose effects [19]. Taken together with gene expression data, these findings suggest that irradiation induced persistent oxidative stress that impaired colony formation via reduced proliferation (e.g., delayed or halted cell division) leading to lower extent of mineralization. Higher doses of γ irradiation (>500 cGy) are required to recapitulate the impairment of osteoblastogenesis [41,42], where changes are also associated with oxidative damage [43]. Taking these findings into consideration, the sequelae of a high-dose of heavy-ion irradiation are likely impaired osteoblastogenesis, which may be attributable to persistent oxidative stress.

For evaluating structural responses, we analyzed cancellous regions in the tibia (Figure 3) and the cortical midshaft of the femur (Table 1) at two time points for parameters indicative of structural integrity. At 5-weeks after irradiation, exposure to high-LET ^56^Fe irradiation at or above 50 cGy caused decrements in fractional bone volume and trabecular number, and increases in trabecular spacing (Figure 3), indicating a significant deterioration of cancellous microarchitecture, consistent with previous findings [20,22,44]. The magnitude of the changes caused by protons or ^56^Fe were similar, although the effect of protons at 50 cGy on BV/TV was not statistically significant (*p* = 0.12). Thus, these findings indicate that the threshold for a persistent, ^56^Fe radiation-induced response lies between 10 and 50 cGy—doses relevant to exploration missions [11]. Overall, we show that a high dose of radiation was able to induce persistent decrements in cancellous bone, regardless of radiation type (Figure 3). Others have shown that these changes persist to four months post-exposure [22]. In this study, the structural decrement affected by ^56^Fe (200 cGy) was resolved one year after exposure due to the expected age-related decline in sham-control animals changes over time (Table 1), likely the product of age-induced elevations of osteoclasts overtaking waning bone formation by osteoblasts [45]. These data are consistent with our previous findings from growing female mice after exposure to low-LET γ radiation [46]. Although others have shown cortical changes following irradiation with either low-LET γ at high doses (500 cGy) [47] or high-LET ^56^Fe at 50 cGy [48], no irradiation-induced cortical responses at 1-year post exposure were evident in this study [23], or in other experiments we have performed in the past. Thus, the influence of ionizing radiation at 200 cGy or below appears to be restricted to the more metabolically-active cancellous tissue.

In vitro irradiation studies were conducted to further assess the role of oxidative stress in radiation damage to osteoprogenitors. Our approach was to test antioxidants and their capacity to rescue radiation effects to provide insight into the different mechanisms between low-LET radiation and high-LET radiation [31]. Although similar doses of low-LET radiation are generally less deleterious than high-LET radiation, the mechanisms for radiation damage to primary, marrow-derived osteoprogenitors in the context of oxidative stress are not fully elucidated [49,50,51]. When comparing the effects of low-LET radiation (^137^Cs) and high-LET radiation (^56^Fe), we found high-LET irradiation was more damaging with respect to growth of the cell population, as assessed by a surrogate assay (changes in DNA content over time). Furthermore, exogenous SOD effectively protected osteoprogenitors from low-LET radiation damage, but only minimally from ^56^Fe at 100 cGy, and not at all from ^56^Fe at 200 cGy. Taken together, these findings indicate excess extracellular reactive oxygen species (ROS) inhibited growth, and that oxidative stress is possibly the main mechanism involved in low-LET radiodamage to osteoprogenitors; in contrast, high-LET species likely affect the cells via additional detrimental mechanisms, such as double-strand DNA breaks.

Although others [52,53] have shown the beneficial effects of various antioxidants to confer radioprotection for various osteoblast cell lines, we show here a direct comparison of antioxidant treatment with different radiation species when added to primary, marrow-derived osteoprogenitors. Interestingly, adding SOD at higher doses or for longer periods was not advantageous, emphasizing the importance of carefully titrating radical scavengers such as SOD, possibly to maintain endogenous ROS-dependent signalling.

In sum, we showed the ability of the antioxidant superoxide dismutase (SOD) to mitigate the adverse effects of in vitro irradiation with γ rays (used for the low-LET radiation), but not to an equivalent dose of high-LET ^56^Fe. Our results strengthen the potential application of SOD as a countermeasure for low-LET irradiation-induced damage to marrow stem cells and osteoprogenitors.

Inherent limitations of these experiments with respect to radiation exposures include: (1) exposures were delivered at high dose-rate and fluence in a matter of minutes, whereas in space, the same dose is delivered at a lower rate, and (2) space exposures are mixed doses, rather than a single species (e.g., SPE can deliver 100–200 cGy over days and GCR over years). In addition, with respect to relevance to spaceflight, these experiments were performed in mice that were normally ambulatory, thus not subjected to musculoskeletal disuse and fluid shifts, which are induced by microgravity; groundbased models for spaceflight have shown that unloading can influence radiosensitivity [17,24,54,55].

In summary, our findings indicate that although low doses of radiation do not show lasting effects on skeletal health, a high dose of high-LET ^56^Fe impairs osteoblast growth and maturation by adversely affecting growth of osteoprogenitors. There were few, select changes in steady-state expression of redox-related genes during osteoblastogenesis ex vivo after total body irradiation with ^56^Fe (but not protons), with some indication of altered expression of select genes during the proliferative phase. Furthermore, scavenging ROS with antioxidants, such as SOD, mitigated the adverse effects of in vitro irradiation with γ rays (200 cGy), but failed to protect from irradiation with an equivalent dose of heavy ions. Together, our findings indicate that high-dose radiation has a persistent effect to impair bone formation by adversely affecting growth, but not differentiation, of osteoprogenitors.

## 4. Materials and Methods

### 4.1. Animals

Male C57BL/6J mice (Jackson Laboratories, Bar Harbor, ME, USA) were individually housed under standard conditions and provided food (LabDiet 5001, Purina, St. Louis, MO, USA) and water *ad libitum* as described by [13]. As a general measure of animal health, we measured body mass weekly for the duration of the study. Additionally, we blindly scored coat color as black or grey at the one-year post-irradiation endpoint. Animals were euthanized by cardiac puncture while under isoflurane overdose followed by cervical dislocation. The Institutional Animal Care and Use Committees for NASA Ames Research Center (Protocol NAS-10-001-Y2, 17 February 2011) and Brookhaven National Laboratory (Protocol #431, 8 February 2011) approved all of the procedures.

### 4.2. In Vivo Experiment Design and Radiation Exposure

Two experiments using mice were conducted to assess the effect of radiation type on skeletal antioxidant response, osteoblastogenesis, and bone structure. In the first experiment (Figure 1A), conscious 16-weeks old, male, C57BL6/J mice were exposed to low-LET protons (150 MeV/n, LET ~ 0.52 keV/µm) or high-LET ^56^Fe ions (600 MeV/n, LET ~ 175 keV/µm) at 5, 10, 50, or 200 cGy at NASA’s Space Radiation Lab at Brookhaven National Lab (Upton, NY, USA). Between 7–9 days after irradiation, mice were shipped overnight from Brookhaven to NASA Ames Research Center, Moffett Field, CA, USA. Select groups of mice were euthanized and tissues harvested after 35–38 days for ^56^Fe, 36–39 days for proton, or 36–38 days for the sham (these three groups are deemed 5 weeks post-irradiation and had *n* = 8 per group) and late (358 or 360 days, here deemed 1 year post-irradiation and had *n* = 13 per group) for ex vivo osteoprogenitor culture of marrow cells. Tibiae or femora were collected for cancellous microarchitecture assessment with microcomputed tomography. For the 5-week endpoint, osteoblast cultures (*n* = 6–7 mice/group) were analyzed by real-time qPCR (*n* = 4 mice/group, see below for details), total DNA quantification, and alkaline phosphatase activity during the proliferative phase (seven days after plating) or the mineralization phase (19–21 days after plating) stages of osteoblast differentiation. At the termination of cultures, mineralized nodules were imaged and analyzed for percent surface area at the end of the culture period. For the 1-year endpoint, osteoblast cultures (*n* = 6/group) were analyzed for colony number during the proliferative phase (eight days after plating) or for mineralized nodule area during the mineralization phase (28 days after plating) of osteoblast differentiation.

In the second experiment (Figure 1B), we aimed to determine the effects of total-body irradiation on the antioxidant capacity of the extracellular fluid surrounding the marrow cells [56]. To evaluate ion effects, conscious 16-wk old, male, C57BL6/J mice mice were exposed to 10 or 200 cGy of low-LET protons (*n* = 6–8 per group) or 5 or 200 cGy of high-LET ^56^Fe ions (*n* = 8 per group) as described above, or were sham irradiated (*n* = 13 per group). Mice were euthanized and tissues harvested at one or seven days after exposure, at which point tibial marrow was processed for extracellular fluid collection and quantification of total antioxidant capacity, described in more detail below.

### 4.3. In Vitro Experiment Design and Radiation Exposure

Osteoprogenitors were grown in vitro from the bone marrow of 16-week old, male C57Bl/6 mice, as previously described [17]. Cells were irradiated three days after plating (day 3) with either γ (^137^Cs, sham or 10–500 cGy, JL Shepherd Mark I) or ^56^Fe (sham or 50–200 cGy, NSRL), and then grown until day 10. Cell behavior was assessed by measuring changes in DNA content between day three and day 10 (as a surrogate for cell growth) with the Cyquant assay as previously described [17]. For the SOD experiment, multiple doses were tested in preliminary experiments (100–600 U/mL of SOD or vehicle, for 2 or 3 days of treatment at different times). The protocol selected for more extensive dose-response experiments entailed the addition of SOD (200 U/mL), or vehicle (α-MEM) 2 h before irradiation (day 3), twice a day, through the 4th day after exposure to radiation. Experiments were repeated 2–3 times. Data were normalized to the sham-control.

### 4.4. Total Antioxidant Capacity

We measured the antioxidant capacity of the extracellular fluid of the marrow (i.e., the marrow plasma) with a colorimetric assay based on the reduction potential of Cu^2+^ to Cu^1+^ as compared to Trolox equivalent standards (Oxford Biomedical, Oxford, MI, USA). In brief, we isolated marrow ex vivo, using 150 µL of sterile saline to flush the marrow from the tibial diaphysis, as described previously [56]. The solution was centrifuged and supernatant preserved as the extracellular fluid at −80 °C for further assessment. The BCA assay assessed the protein concentration of the extracellular fluid against albumin standards, which enabled the normalization of the antioxidant capacity by the protein concentration to account for changes in protein levels with treatment [56].

### 4.5. Bone Microarchitecture and Geometry

We assessed the bone volume and microarchitecture of the cancellous tissue in the proximal tibial metaphysis using microcomputed tomography (6.7 µm pixel size, 3500 ms integration time, 50 kV, Skyscan 1174; Bruker microCT, Kontich, Belgium) as described in [13]. For cancellous quantification, a 1.0-mm thick region located 0.24 mm distal to the proximal growth plate was semi-autonomously contoured to include cancellous tissue. We assessed changes in cancellous bone using the three-dimensional bone volume to total volume fraction (BV/TV, %), trabecular thickness (Tb.Th, mm), trabecular number (Tb.N, 1/mm), trabecular thickness (Tb.Th, mm), and trabecular separation (Tb.Sp, mm). We assessed the changes in cortical bone at the midshaft of the femur using bone volume and cortical thickness. All analyses follow conventional guidelines [57].

### 4.6. Ex Vivo Osteoblastogenic Assays and qRT-PCR Analyses

For osteoblastogenic culture, femur marrow was flushed using saline and treated with lysis buffer specific for red blood cells (Sigma-Aldrich, St. Louis, MO, USA), centrifuged for ten minutes at 1000× *g*, and supernatant removed. Cells were then re-suspended and plated at 3.0 × 10^5^ cells/cm^2^ in 6-well plates. Osteoblastogenic growth medium (alpha minimum essential medium with 15% FBS (Gibco, Gaithersburg, MD, USA), 1× anti-biotic/mycotic, 50 µg/mL ascorbic acid, 10 mM β-glycerophosphate) was replenished every 2–3 days.

For qPCR analyses (*n* = 4/group), cells were collected in a solution of 1% β-mercapthoethanol in RLT Buffer (Qiagen, Valencia, CA, USA) and stored at −80 °C until RNA isolation via RNeasy minikit (Qiagen). RNA quantity and purity was measured using a nanodrop spectrophotometer (Nanodrop 2000, Thermoscientific, Waltham, MA, USA) while RNA quality was assessed using a 0.8% non-denaturing gel. A RT^2^PCR custom array (PAMM-999A-1, SYBR probes, Qiagen, Hilden, Germany) was processed with an Applied Biosystems 7500 cycler to generate gene expression data. The ΔΔ*C*_t_ method was used to analyze the raw data. Cycle values above 35 were defined as non-detectable. Statistical analyses were performed on log_2_-transformed data. Fifteen representative genes were analyzed to assess osteoblast differentiation, proliferation, oxidative metabolism, or apoptosis, see Table 3. All genes were normalized to housekeeping gene *Hprt*. Technical issues forced the loss of one specimen from the ^56^Fe, 200 cGy group during the proliferative phase and the loss of all four specimens in the proton, 10 cGy group from mineralization phase.

For ALP and DNA analyses, cells were collected in a solution of Tris buffer containing Triton-X and MgCl_2_ and stored at −80 °C. Quantification of ALP enzymatic activity and total DNA content was performed using Alkaline Phosphatase Activity Colorimetric Assay (Biovision, Mountain View, CA, USA) and CyQuant Cell Proliferation Assay Kit (Thermo Fisher Scientific, Waltham, MA, USA), respectively.

Colony counts early in culture assessed the successful adherence and proliferation of colony forming units. Parameters defining a colony included having distinct region of radial colony growth and having more than 30 cells with a majority of cells having of morphological homogeneity.

To quantify densely mineralizing area (i.e., the nodule area), we harvested cultures in the differentiation phase. First, we washed cultures with saline and scanned the plates at 300 dpi resolution. We quantified the percentage of well area having mineralizing nodules with Image J, using a custom algorithm to threshold the grayscale image.

### 4.7. Statistics

We report parametric data with mean and standard deviation (SD). For these data, to determine significant differences, a 1-way analysis of variance was used followed by Dunnett’s post-hoc test, which allowed for the comparison of respective radiation types and doses to the age-matched sham control (0 cGy). For non-parametric data (i.e., nodule area data, as values near zero display skew), we report the median with interquartile range. For these data, to determine significant differences, we used a Kruskal-Wallis test with a Steel post-hoc test, which allowed for the comparison of respective radiation types and doses to the age-matched, sham control (0 cGy). We assessed temporal (within subjects) treatment (between subjects) effects in body mass, and their interaction (i.e., time × treatment), using Repeated Measures ANOVA and Wilks’ Lamda test. Throughout, a *p*-value ≤ 0.05 was accepted as significant. We performed all analyses using JMP 13 (SAS).

## Figures and Tables

**Table 1:** Fur coat color and cancellous microarchitecture of the proximal tibial metaphysis 1 year after ^56^Fe irradiation.

Parameter	Sham	10 cGy	50 cGy	200 cGy
Grey/Not Grey Coat (%)	1/12 (7.7%)	1/12 (7.7%)	1/11 (8.3%)	11/0 (100%)
Percent Bone Volume (%)	9.7 ± 3.1	8.7 ± 2.4	8.2 ± 2.9	7.2 ± 1.5
Trabecular Thickness (μm)	59.3 ± 5.3	58.2 ± 3.2	57.7 ± 2.9	57.1 ± 2.7
Trabecular Separation (μm)	228.8 ± 23.3	245.0 ± 41.8	240.1 ± 30.2	248.5 ± 18.4
Trabecular Number (1/mm)	1.62 ± 0.36	1.49 ± 0.35	1.42 ± 0.48	1.26 ± 0.23

**Table 2:** List of genes quantified from ex vivo culture, which did not show significant changes in expression levels. Cells were isolated and cultured five weeks post-irradiation and analyzed at proliferative and terminally differentiated stages in growth.

Proliferative Stage	Terminal Differentiation
*Caspase 3*	*Caspase 3*
*Cdk2*	*Catalase*
*CuZnSOD*	*Cdk2*
*Foxo1*	*Gadd45*
*GPX*	*iNos*
*MnSOD*	*MnSOD*
*p21*	*p53*
*p53*	*PCNA*
*PCNA*	-
*Runx2*	-

**Table 3:** SYBR-based custom array to query changes in expression levels of key genes.

Gene Name	Accession No.	Official Name	Process
*Runx2*	NM_009820	Runt Related Transcription Factor 2	Differentiation
*Bglap*	NM_007541	Bone γ carboxyglutamate protein (osteocalcin)	Differentiation
*Alpl*	NM_007431	Alkaline Phosphatase Ligand	Differentiation
*Pcna*	NM_011045	Proliferating Cell Nuclear Antigen	Proliferation
*Cdk2*	NM_016756	Cyclin-dependent Kinase 2	Proliferation
*p21*	NM_007669	Cyclin-dependent Kinase Inhibitor 1	Proliferation
*p53*	NM_011640	Transformation-related protein 53	Proliferation
*Cat*	NM_009804	Catalase	Oxidative Metabolism
*Gpx1*	NM_008160	Glutathione Peroxidase	Oxidative Metabolism
*MnSod*	NM_013671	Superoxide Dismutase 2, Mitochondrial	Oxidative Metabolism
*CuZnSod*	NM_011434	Superoxide Dismutase	Oxidative Metabolism
*iNos [Nos2]*	NM_010927	Nitric Oxide Synthase 2, Inducible	Oxidative Metabolism
*Foxo1*	NM_019739	Forkhead Box	Oxidative Metabolism
*Gadd45a*	NM_007836	Growth Arrest and DNA Damage	Oxidative Metabolism
*Caspase 3*	NM_009810	Caspase 3	Apoptosis
*Hprt*	NM_013556	Hypoxanthine-guanine phosphoribosyltransferase	Housekeeping
